# Point-of-care system for rapid real-time detection of SARS-CoV-2 virus based on commercially available Arduino platforms

**DOI:** 10.3389/fbioe.2022.917573

**Published:** 2022-08-04

**Authors:** Huynh Van Ngoc, Than Linh Quyen, Aaydha Chidambara Vinayaka, Dang Duong Bang, Anders Wolff

**Affiliations:** ^1^ BioLabChip Group, Department of Biotechnology and Biomedicine, Technical University of Denmark (DTU-Bioengineering), Lyngby, Denmark; ^2^ Laboratory of Applied Micro and Nanotechnology (LAMINATE), Department of Biotechnology and Biomedicine, Technical University of Denmark (DTU-Bioengineering), Lyngby, Denmark

**Keywords:** point-of-care, fluorescence detection, SARS-cov-2, COVID-19, loop-mediated isothermal amplification, lab-on-chip, open-source software-hardware, rapid diagnostic

## Abstract

The COVID-19 pandemic emphasized the importance of rapid, portable, and on-site testing technologies necessary for resource-limited settings for effective testing and screening to reduce spreading of the infection. Realizing this, we developed a fluorescence-based point-of-care (fPOC) detection system with real-time reverse transcriptase loop-mediated isothermal amplification for rapid and quantitative detection of the severe acute respiratory syndrome coronavirus 2 (SARS-CoV-2) virus. The system is built based on the Arduino platform compatible with commercially available open-source hardware–software and off-the-shelf electronic components. The fPOC system comprises of three main components: 1) an instrument with integrated heaters, 2) optical detection components, and 3) an injection-molded polymeric cartridge. The system was tested and experimentally proved to be able to use for fast detection of the SARS-CoV-2 virus in real-time in less than 30 min. Preliminary results of testing the performance of the fPOC revealed that the fPOC could detect the SARS-CoV-2 virus at a limit of detection (LOD_50%_) at two to three copies/microliter (15.36 copies/reaction), which was comparable to reactions run on a standard commercial thermocycler. The performance of the fPOC was evaluated with 12 SARS-CoV-2 clinical throat swab samples that included seven positive and five negative samples, as confirmed by reverse transcription–polymerase chain reaction. The fPOC showed 100% agreement with the commercial thermocycler. This simple design of the fPOC system demonstrates the potential to greatly enhance the practical applicability to develop a totally integrated point-of-care system for rapid on-site screening of the SARS-CoV-2 virus in the management of the pandemic.

## 1 Introduction

The concept of point-of-care (POC) testing originated in the 15th century (Nguyen et al., 2020); however, because of the high demands from clinical diagnostic markets, modern POC diagnostics emerged in the last decades (Mahmoudi et al., 2020; 2021a). A POC device that is robust, portable, sensitive, cost-effective, easy to handle, and offering on-site rapid sample-to-answer results can be used in resource-limited and non-laboratory settings and can also be operated by non-technical operators with minimal laboratory training (Nguyen et al., 2020). To date, the POC testing is developed based on an antibody/sera test, antigen test, and molecular test and applied in different fields from clinical diagnostics to food safety and environmental monitoring (Mahmoudi et al., 2021b; Koteswara Rao, 2021; Mahmoudi et al., 2022).

The COVID-19 pandemic is caused by a new severe acute respiratory syndrome coronavirus 2 (SARS-CoV-2). According to the World Health Organization (WHO), by the 20th of February 2022, the COVID-19 pandemic affected 220 countries, areas, and territories with more than 492 million confirmed cases and more than 6.1 million deaths (https://covid19.who.int/). The pandemic not only demonstrated a severe effect on health but also caused social turmoil and economic disruption (https://www.ilo.org/wcmsp5/groups/public/---ed_dialogue/---act_emp/documents/publication/wcms_745024.pdf; https://www.un.org/development/desa/dspd/; https://en.unesco.org/covid19/educationresponse; https://www.worldbank.org/en/news/factsheet/2020/07/13/economic-and-social-impacts-of-covid-19-update-from-listening-to-tajikistan; https://en.unesco.org/sites/default/files/issue_1_en_culture_covid-19_tracker.pdf). Researchers showed that the disease spreads easily through contact, droplets, airborne, fomite, fecal–oral, mother-to-child, and animal-to-human (https://www.who.int/news-room/commentaries/detail/transmission-of-sars-cov-2-implications-for-infection-prevention-precautions). Preventing the transmission of the disease will remarkably reduce the negative effects on the health, economy, and society.

During the COVID-19 pandemic, reducing the rate of spreading of the infection was difficult. Therefore, WHO recommended to increase the frequency of testing as well as to identify, isolate, and quarantine the positive cases (https://apps.who.int/iris/handle/10665/340072). Next, reverse transcriptase polymerase chain reaction (rRT-PCR) and later on antigen-based lateral flow tests were implemented as officially approved methods to detect SARS-CoV-2 in the clinical samples. However, the rRT-PCR is a time-consuming assay that requires sophisticated laboratory facilities, well-trained personnel, and the performance may be influenced by inhibitory effect (https://altona-diagnostics.com/en/products/reagents-140/reagents/realstar-real-time-pcr-reagents/realstar-sars-cov-2-rt-pcr-kit.html). Another major drawback of rRT-PCR is its high cost/assay that was beyond the reach of resource-limited countries (Aziz et al., 2020; Giri and Rana, 2020). On the other hand, many antigen-based quick tests do not reach sensitivity required to detect the early phase of SARS-CoV-2 infection (https://ec.europa.eu/health/sites/health/files/preparedness_response/docs/common_testingapproach_covid-19_en.pdf). For example, major lateral flow based quick testing kits such as Panbio™ COVID-19 Ag Rapid Test Device from Abbott (https://ec.europa.eu/health/sites/health/files/preparedness_response/docs/common_testingapproach_covid-19_en.pdf) claim >99% positive agreement in the clinical sensitivity with the samples demonstrating Ct values ≤ 33 (that could be equivalent to ∼60–70 copies/μL as determined by rRT-PCR standards) with a limit of detection (LOD) at ∼0.25 TCID_50_/μL of SARS-CoV-2 with a clinical sensitivity of 91.4%. All of these have become bottleneck in the rapid surveillance of SARS-CoV-2 and effective management of the pandemic. Loop-Mediated Isothermal Amplification (LAMP) has been demonstrated as a powerful alternative to overcome the drawback of PCR (Rahman et al., 2017; Kaur et al., 2018; Rabe and Cepko, 2020; Lalli et al., 2021). Next, LAMP is an isothermal amplification technique and can perform at a constant temperature between 60°C and 65°C. LAMP exhibits several advantages e.g., high specificity, high sensitivity, fast amplification, and more resistance to inhibitors than PCR (Notomi, 2000; Nagamine et al., 2002; Francois et al., 2011; Njiru, 2012; Gotoh et al., 2013; Stedtfeld et al., 2014; Kostić et al., 2015; Velders et al., 2018; Lalli et al., 2021). Moreover, the LAMP reaction produces a huge amounts of amplified product (dsDNA) and by-product (magnesium pyrophosphate) that can easily be detected by several different detection methods and even by naked eyes (Amin Almasi et al., 2013; Xie et al., 2014; Tanner et al., 2015; Dao Thi et al., 2020; Vinayaka et al., 2022). With these advantages, the LAMP may be an alternative to PCR in rapid POC diagnostic applications.

To date, researchers reported several POC devices for detection of SARS-CoV-2 (Soares et al., 2021; Torezin Mendonça et al., 2022; Trick et al., 2022). These POC devices demonstrated a simple design and fast detection. However, fabrication of the test cartridge resulted in complicated approaches such as laser ablation process (Torezin Mendonça et al., 2022), laser-cutting coupled with thermoforming (Chen et al., 2020), and soft lithography (Deng et al., 2021) that are difficult to scale-up at low-cost. Moreover, the number of samples tested per run was limited (one sample per run), such as in the disposable electrochemical sensing chips (Chen et al., 2020; Deng et al., 2021; Rodriguez-Manzano et al., 2021; Beduk et al., 2022; Kaci et al., 2022). Also, the electrochemical sensing chips are susceptible to interferences from charged matrices as well as biological materials, such as sputum and cough, that may generate fouling effect. In this work, we report a point-of-care detection system developed based on fluorescence optical module (fPOC) and LAMP technology that can overcome these drawbacks of current POCs: 1) testing cartridges demonstrate a simple design that are cost-effective and fabricated by injection molding, which is feasible for fast mass production; and 2) in the fPOC, scaling up the number of tested samples per run is possible. The developed fPOC is suitable for on-site real-time rapid detection of the SARS-CoV-2 virus in clinical diagnostics and for mass surveillance of SARS-CoV-2. A real-time reverse transcriptase loop-mediated isothermal amplification (rRT-LAMP) assay to detect SARS-CoV-2 was adapted to the fPOC system, and the analytical performance of the fPOC was compared with commercial lab-bench qPCR system and an *in-house* developed turbidity-based POC detection system.

## 2 Materials and methods

**Electronic components**: The electronic components of the fPOC system are based on open-source hardware–software of Arduino platforms (https://www.adafruit.com/; https://www.arduino.cc/) and off-the-shelf electronic components (https://dk.farnell.com/) https://dk.rs-online.com/). Supplementary Table S1 , in the Supplement Information, shows a list of components used in the system and suppliers.

**Light sources**: Five different blue light emitting diodes (LED) demonstrating a center emitting wavelength at 470 nm, with different viewing angle and maximum luminous intensity, were used as the light sources to optimize the excitation light (C503B-BCN-CV0Z0461, L-19804VBC/DS-D, NSPB500AS, HLMP-CB1B-XY0DD, C503B-BAN-CZ0A0451).

**Filters**: Two sets of excitation (EX) and emission (EM) filters, 1) Omega filters 465DF10–465 nm and 510DF10–510 nm with bandpass of 10 nm; https://www.omegafilters.com/optical-filters/bandpass and 2) Optolong filters 480 nm and 530 nm with bandpass of 30 nm, http://www.optolong.com/) were used to collect and extract the required excitation and emission fluorescence light wavelengths, respectively.

**Heaters**: Two heating elements from Keenovo silicone heaters (www.keenovo.com), 60 mm × 60 mm in size, at power of 50 W @ 30 V, were used to build the heater system. The heating elements were stalked as such that the polymeric cartridge is sandwiched between top and bottom heating elements. Next, the top heater demonstrated custom made holes Ø of 5 mm under EX and EM filters in order to let the excitation light go through the sample and fluorescence emission light back to the detector.

Parallax Data Acquisition tool (PLX-DAQ), an open access freely downloadable software) run on an external computer, was used to receive and process the data sent from the system (https://www.parallax.com/package/plx-daq).

### 2.1 Reverse transcriptase real-time LAMP reaction

LAMP primer set targeting gene N (nucleocapsid phosphoprotein) of SARS-CoV-2 was selected for this study (Zhang et al., 2020). The rRT-LAMP assay was carried out in 30 μL LAMP master mixture containing 0.2 μM of F3, 0.2 μM of B3, 1.6 μM of FIP, 1.6 μM of BIP, 0.8 μM of LF, 0.8 μM of LB (Integrated DNA Technologies, Leuven, Belgium), 1.4 mM dNTP mix (Thermo Fisher Scientific, Roskilde, Denmark), 0.25 M Betaine (Sigma-Aldrich, Denmark), 1.5 mM MgSO_4_, 9U of Warmstart^®^ RTx Reverse Transcriptase, 12 U of *Bst* Warmstart^®^ 2.0 DNA polymerase, 1× isothermal amplification buffer (New England BioLabs), 1.67 µM of SYTO-9 (Invitrogen), sterilized water, and 6 μL template.

The rRT-LAMP reactions were performed in parallel in different systems that include the developed fPOC system: an *in-house* developed turbidity-based POC device - the PATHPOD system (Bang et al., 2021), a commercial lab-bench qPCR systems (Stratagene Mx3005P, AH Diagnostic, Denmark), and PIKO qPCR (Thermo Fisher Scientific, Finland). Further, the rRT-LAMP assay was performed at 65°C for 50 min. Next, the reaction was terminated by increasing the temperature to 90°C for 5 min.

### 2.2 Plasmid control

Commercial 2019-nCoV_N_Positive control contained the completed nucleocapsid gene of 2019-nCoV were purchased from Integrated DNA Technologies (Leuven, Belgium) and used as positive control.

To estimate the LOD and limit of quantification (LOQ), a log_5_ dilution of plasmid control ranging from 48,000 to three copies per reaction was analyzed in three replicates. Negative oral swab sample without any target was tested in replicates of 3 as the negative control (NC). The threshold time (T_t_) was recorded for NC that was at 32.76 T_t_. The lowest concentration (76.8 copies/reaction) that gave positive results in all three repetitions was considered LOD of the system with 100% confidence level. Next, target concentration at 15.36 gave partial positive results, with two out of four reactions showing positive results, and that was considered as LOD_50%_. LOQ was estimated by adding the average T_t_ value at LOD_50%_ (21.32) and the standard deviation (2.32) of the lowest concentration tested with a 100% confidence level.

### 2.3 Clinical samples

Throat swab samples from voluntary individuals were collected at Hvidovre hospital, Denmark during the COVID-19 pandemic. The RNA from the samples were extracted and purified by MagNA pure 96 DNA and viral NA small volume kit (LifeScience, Roche, Denmark), following the instruction of the manufacturer. Six µL of the extracted solution were used as template in the rRT-LAMP reaction.

## 3 Results and discussion

### 3.1 Design and fabrication of fluorescence-based point-of-care device

#### 3.1.1 fPOC working mechanism

**FIGURE 1:**
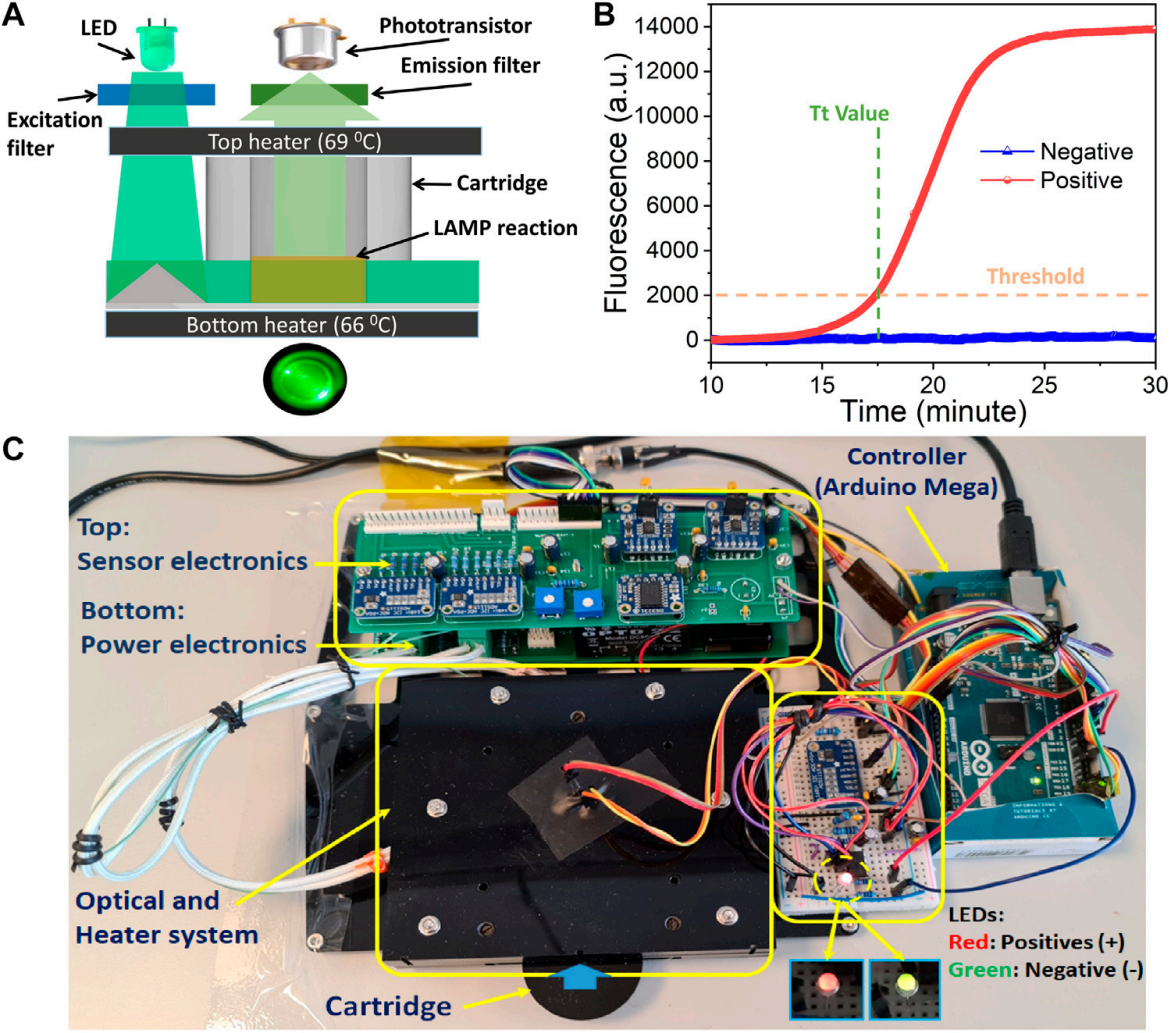
fPOC system: **(A)** fluorescence detection and operation principle of the system, and **(B)** fluorescence signal intensity with control negative and positive samples, and **(C)** a fPOC prototype.

The fPOC working principle (Figure 1A) is based on three main elements that includes the following: 1) an injection molding cartridge with 12 reaction wells (Bang et al., 2021). In this study, an optical reader was set up for one well in the fPOC prototype; however, it can be easily extendable to all 12 wells and can even be extended to 16, 96 wells or more as required by the end-user. Also, the cartridge contains specially designed pyramid shaped optical structure located next to the reaction wells (Supplementary Figure S1) for reflecting the LED light to the reaction well at 90° angle, 2) two heating elements (top and bottom heaters) to maintain the temperature (60°C–65°C) for the rRT-LAMP reaction, and 3) optical elements that include a light source (LED), a phototransistor, and EX/EM filters.

The well is heated rapidly and maintained at the reaction temperature (60°C–65°C) by the two heaters: bottom and transparent top heater, respectively (Figure 1A). The targeted nucleic acids (RNA/DNA) in the sample were amplified by rRT-LAMP within the well. The generated rRT-LAMP-amplified products were fluorescence labeled by the intercalation of SYTO-9 DNA intercalating dye. The blue LED light (470 nm) shined on top of the pyramid shaped optical structure was reflexed by total internal reflection at a right angle through the well. This excites the intercalated SYTO-9 fluorophore dye in the amplified rRT-LAMP products. To measure the fluorescence light emitted from the reaction well, a phototransistor is placed above the chamber. Next, a set of excitation and emission filters is used to separate the excitation and emission wavelength of the spectrum. The fluorescence signals are recorded every second during the reaction time and plotted in real-time using the Parallax Data Acquisition tool (PLX-DAQ) software on an external computer (Supplementary Figure S2A). The baseline of the signal from phototransistor is obtained at the 10th minute. When the reaction is completed, based on the threshold signal values, the samples are defined as positive or negative. The sample is considered positive when the fluorescence intensity is larger than the threshold signals (Figure 1B). Next, these thresholds were determined after performing a series of NC experiments on the system. In the stand-alone mode (without computer/laptop), the test results are displayed on the device with two-color LEDs showing the sample as positive when the LEDs are in red color blinking, while they are negative when the LEDs are in green color (Figure 1C).

### 3.2 System design and detection optimization

The design and development of fPOC system (Figure 1C) was based on an *in-house* developed POC device (PATHPOD) that monitored the LAMP reaction by turbidity. The turbidity detection principle of PATHPOD is based on the variation in the light transmittance as a result of precipitation of the magnesium pyrophosphate formed during the amplification of DNA. Next, green light (535 nm) is sent from a LED through the sample, and a phototransistor is used to measure the intensity of transmitted light (Bang et al., 2021).

The 3D image of the reaction well is shown in Supplementary Figure S1 (Supplementary Information). The well is made of Topas 5013L-10 cyclic olefin copolymer (COC) material by polymer injection molding (Engel Victory 80/45 Tech, York, PA). The well exhibits a diameter of 4 mm and a height of 7.5 mm. The pyramid-shape optical structure with an angle of 45^ο^ is embedded next to the reaction well, which demonstrated a refractive index of COC n_p_ = 1.53. The critical angle of the two media, COC/air, is calculated as 40.8^ο^ (Nguyen et al., 2019). Therefore, the excitation light from the blue LED located on the top of the pyramid is reflected due to total internal reflection at 90° into the reaction well. The design of one pyramid with four sides of optical structure can reflect excitation light to four wells simultaneously, leading to simplification of the chip design and reduction of the required number of light sources (LEDs) and EX filters. In addition, all the optical parts (LED, filter, and phototransistor) located on the top of chip that will increase the total contact of the heater to the bottom of the reaction well can enhance the heat transfer efficiency of the bottom heater to the reaction well. Also, such design opens an opportunity for more integrated different components, for example, sample preparation to develop a total integrated fPOC system for analyzing multiple samples (12, 16, 32, etc. samples).

Schematic diagram of the electronic circuit board of the fPOC system with a single sample detection that was designed based on Arduino platforms is shown in Supplementary Figure S3 (Supplement Information). In general, Arduino platforms are open-source hardware compatible with many available software libraries. This will provide an easy way for novices and professionals to create devices at low-cost using sensors and actuators (https://spectrum.ieee.org/the-making-of-arduino).

Moreover, the PLX-DAQ is a freely downloadable software. The software enabled the computer to receive and process the data generated in the fPOC system. Next, PLX-DAQ is an add-on tool for Microsoft Excel (https://www.parallax.com/package/plx-daq) that allows transferring of collected data from Arduino-Controller board Mega 2,560 (such as fluorescence intensity, temperature, and time) to a PC and processing directly in excel format. Then, the received data can be processed, plotted, or graphed, as well as read/write on a worksheet in real-time using Microsoft Excel.

The heaters used to maintain the temperature precisely at 65°C for the rRT-LAMP reaction included a 50 W @ 30 V silicone heater, an FQP30N06L MOSFET power transistor that controlled the current through the heater, and an Arduino-based Adafruit MAX31855 Temperature Sensor Development Tools. A DC60MP solid state relay was used as a safety-lock in the system that protected the heater from a possible overheating. The relay functioned by switching off the supply of current to the heaters when the temperature of one of the heaters exceeds 100°C. To maintain the reaction temperature precisely at 65°C inside the reaction well, the local temperatures of individual heaters were set at 69^o^C and 66^o^C for the top and bottom heaters, respectively. The set up temperature with the accuracy of ±0.25^o^C was controlled by a PID control algorithm. Next, in order to avoid the condensation of vapor on the sealing film, the temperature of the top heater was set 3°C higher than the bottom heater (Supplementary Figure S2B). For optical detection module of the fPOC system, we used a blue LED L-19804VBC/DS-D as an excitation light source that demonstrated an emission center wavelength at 470 nm and an Infrared/Visible phototransistor Vishay BPW77NA for collecting the fluorescence light emitted from the sample in the well. To extract the required excitation and emission fluorescence light wavelengths, a set of Omega filters that includes 465DF10 (465 nm center wavelength and 10 nm bandpass) and 510DF10 (510 center wavelength and 10 nm bandpass) filters are used as excitation and emission filters, respectively. The bandpass of the filter sets was aligned with excitation/emission spectrum of SYTO-9 dye used in the rRT-LAMP reaction.

The output analog signal from the phototransistor, presenting as intensity of fluorescence light in terms of photon counts emitted from the sample, was transferred to Arduino-based ADS1115 16-Bit ADC that converted the analog signal to a digital signal. Next, to reconstruct the fluorescence light intensity, this digital signal was processed in the computer.

**FIGURE 2:**
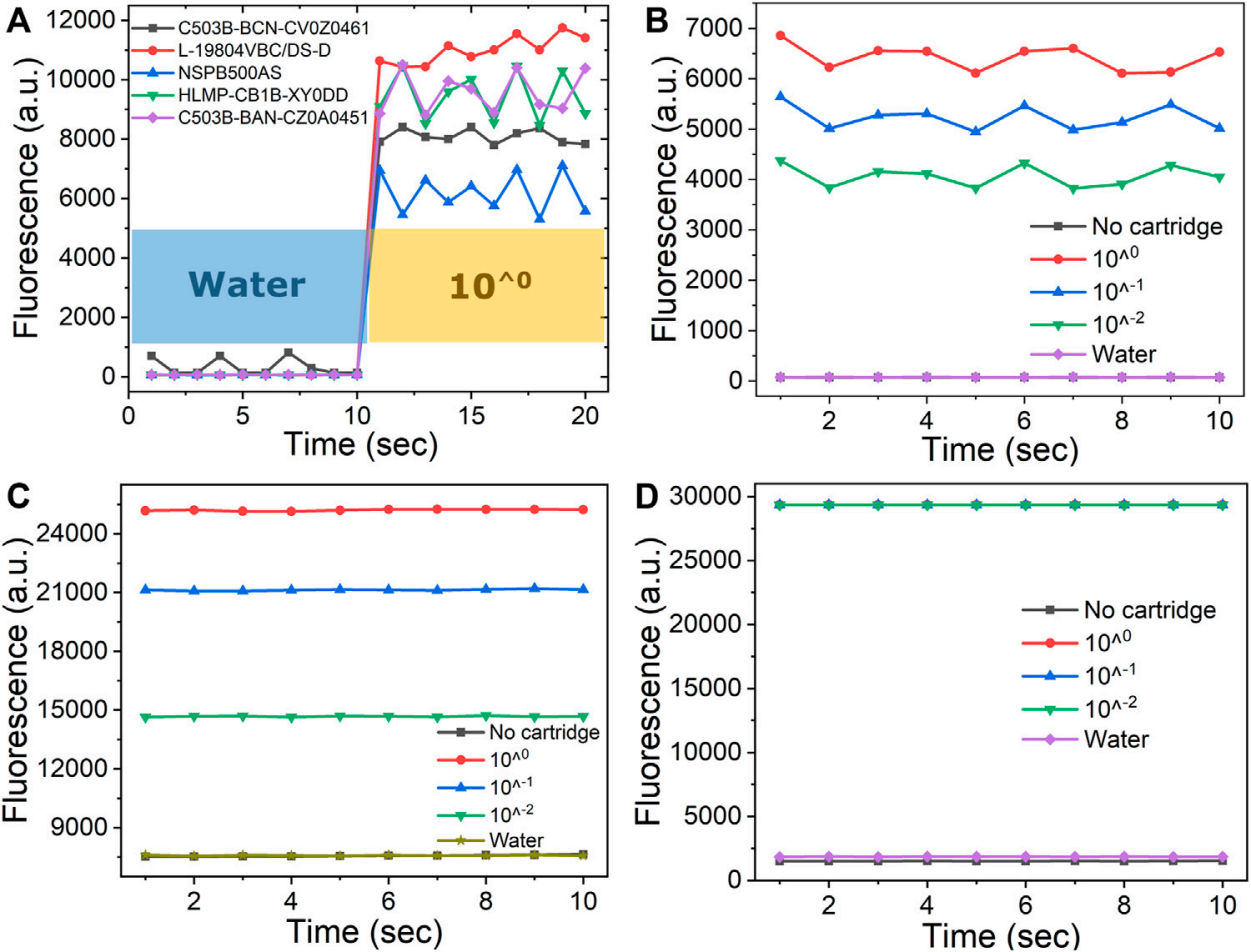
Optimization of fluorescence detection of the fPOC: **(A)** five different blue LEDs as the light sources were tested with water and 10 times dilutions of rRT- LAMP product; the fluorescence intensity before **(B)** and after **(C)** implement with Operational Amplifier tested with water, no cartridge, and a serial dilutions of LAMP product ranging from 10^0^, 10^−1^, 10^−2^; using two different EX/EM filter sets of **(C)** omega and **(D)** Optolong Optics filters.

To optimize the fluorescence detection capacity of the fPOC system, different commercially available blue LEDs, EX/EM filter sets were selected and tested together with an Operational Amplifier (Op-amp). In early initiated experiments, five different blue LEDs at different center of emitting wavelength, emitting angle, and maximum luminescence are selected and tested (Supplementary Table S2 in the Supplement Information). Figure 2A shows that among the five commercially available LED sets, highest fluorescence signal intensity was obtained with the L-19804VBC/DS-D LED. However, the output signals were still low. To improve the output signal, a TLV2374IN Op-amp was used to enhance the received output signals in the ADS1115 ADC from 100 mV to 3 V. The 3 V output values are now in the detection dynamic range of the ADS1115 ADC (Figure 2C). To further optimize the fPOC optical detection module, another set of EX/EM Optolong Optics filters with a 480 nm center wavelength (30 nm bandpass) and 530 nm center wavelength (30 nm bandpass) were used. As shown in Figure 2D, these filters not only improved the intensity of the output signals, thereby saturating the sensor at all the three dilutions of LAMP products tested, but also reduced the background signals as observed with water and in the absence of the well. As a result, the S/N is increased.

To determine the sensitivity of fPOC system, a serial 10-fold dilution of a rRT -LAMP product ranging from 10^0^, 10^−1^, 10^−2^, and water (containing no DNA product that was used as background signal) in presence of 5 µM of SYTO-9 dye was used. The results showed that the rRT -LAMP product at 10^−2^ dilution demonstrated fluorescence intensity of 29,350 arbitrary unit in comparison to 1,850 arbitrary unit observed with the water (Figure 2D). The 29,350 arbitrary unit was the highest signal intensity measured in the fPOC and considered as the saturation point of the detector of the fPOC system. The rRT-LAMP products demonstrating higher concentrations than 10^−2^ dilutions gave similar fluorescence counts due to saturation of the photodetector. Similar observations were made in a commercial lab-bench PCR system Mx3005P. Fluorescence detector got saturated at 6.5 × 10^9^ arbitrary unit observed with the concentrations of the rRT-LAMP product at 10^−2^ dilution, whereas a background signal of 5.2 × 10^8^ unit was obtained for the water (Supplementary Figure S4B). However, using another commercial qPCR system, the PIKO qPCR system, a highest signal intensity measured at 10^0^ dilution of the rRT-LAMP product was determined as 67,518 arbitrary unit, followed by 10^−1^ and 10^−2^ dilutions being 64,121 and 47,490 arbitrary unit, respectively. The lowest signal intensity of 560 arbitrary unit was observed with water (Supplementary Figure S4A). This observation is similar to the results of fPOC system when using Omega filter set (Figure 2C). Therefore, we can safely conclude that the sensitivity of the detector in fluorescence intensity of the fPOC was better than that of the PIKO system and comparable to the lab-bench PCR system Mx3005P.

## 4 Applications

### 4.1 Optimization of DNA dye concentration (SYTO-9) in rRT-LAMP reaction on the fPOC system

To optimize concentration of DNA dye for the rRT-LAMP reaction, SYTO-9 of 1–10 µM (final concentration) were tested in the fPOC system with two different optic filter sets: the Omega and the Optolong Optics filters. In positive reactions, the fluorescence intensity increased quickly after 10 min of the rRT-LAMP reaction, while it remained unchanged in the negative reactions with both filter sets (Supplementary Figure S5). However, the detector reached saturation at 10 µM of SYTO-9 with the Omega filter set (Supplementary Figure S5A), while it was saturated at around 2 µM of SYTO-9 with the Optolong filter set (Supplementary Figure S5B). Therefore, 1.5 µM of SYTO-9 was selected for further investigation of rRT-LAMP assays in the fPOC system. In all the tests, the fluorescence intensity was initially high at the starting point (13,000–20,000 arbitrary unit when using Omega filter set or 6,000–29,000 arbitrary unit when using Optolong filter set). This initial high fluorescence signals that the starting point of the reaction could be due to the formation of hairpin structures of primers at room temperature. However, as the rRT-LAMP reaction mixture reaches the amplification temperature (65°C), the fluorescence signals reach baseline quickly.

### 4.2 Limit of detection of the fPOC system

**FIGURE 3:**
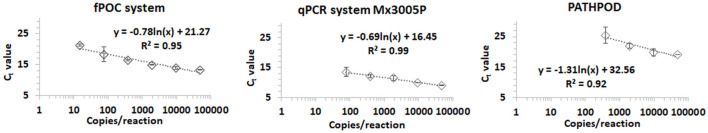
The standard calibration curve of fPOC system, qPCR system Mx3005P, and PATHPOD turbidity-based system using a serial 5-fold dilution of plasmid control as template.

The performance of the fPOC system was further investigated using rRT-LAMP assay with the SARS-CoV-2 plasmid positive control as amplification template. The performance of the fPOC was tested and compared to a commercial PCR thermocycler Mx3005P system and PATHPOD, an *in-house* POC system (mentioned above). A serial 5-fold dilution of the plasmid control demonstrating a starting concentration of 48.000 copies/reaction was prepared and used as a target for the assay. Linear curves were observed in all three tested systems for the serial dilutions of plasmid control (Figure 3). A LOD of 76 copies/reaction was observed in both the fPOC system (T_t_ 18.34 ± 2.32 min) and qPCR Mx3005P system (T_t_ 13.50 ± 1.54 min). However, sensitivity of the turbidity-based PATHPOD system was 5 times less sensitive than the two other systems. The T_t_ value of the PATHPOD system (turbidity detection) was also delayed (T_t_ 25.46 ± 2.76 min), comparing to the fPOC system (T_t_ 18.34 ± 2.32 min) and the lab-bench commercial qPCR Mx3005P system (T_t_ 13.50 ± 1.54 min). The fPOC could detect the SARS-CoV-2 virus at a limit of detection (LOD_50%_) at 15.36 copies/reaction (two to three copies/microliter) and LOQ at 23.6 copies/reaction (∼4 copies/microliter). The results revealed that the performance of the fPOC system is comparable to the commercial thermocycler qPCR Mx3005P system (Stratagene, AH diagnostics, Denmark).

### 4.3 Evaluation the performance of the fPOC system to detect SARS-CoV-2 in clinical samples

Further, we further the performance of the fPOC for detecting of SARS-CoV-2 using 12 clinical throat swabs samples that included seven SARS-CoV-2 positive and five negative samples, as confirmed by RT-PCR (see Materials and Methods section). The performance of the fPOC was compared to the PATHPOD (turbidity-based) and PCR Mx3005P. Of 12 samples tested, seven samples showed a positive signal, and five samples showed a negative signal in the fPOC platform, PATHPOD system, and qPCR system Mx3005P. The performance of the fPOC platform was in 100% agreement with the commercial PCR MX3005P system, the PATHPOD, as well as with the standard rRT-PCR. Besides purified RNA samples, samples were processed by a simple boiling method and could be directly loaded into the testing cartridges without any additional sample purification steps that minimizes possibility of contamination (Dao Thi et al., 2020; Quyen et al., 2022). As a result, it could make the assay field applicable for on-site preliminary quick testing at low-resource settings and reduce the analysis time.

In current times, rRT-LAMP based on both turbidity detection and fluorescence is in practice, since they can monitor the specific gene expression or concentration of target (Mori et al., 2004; Cai et al., 2008; Chen and Ge, 2010; Tourlousse et al., 2012; Cao et al., 2015). A commercial device is available (Eiken Chemical, Co., Ltd., Japan) for monitoring rRT-LAMP amplification. However, sensitivity of the turbidity-based detection method is 10 times lesser than that of the fluorescence detection (Aoi et al., 2006; Chen and Ge, 2010). A commercial fluorescence-based system for detecting LAMP reaction exists that could be used at POC scenarios. However, the system is sophisticated, and the cost of the device is very high (https://www.pro-lab-direct.com/product-p/gen2-01.htm.). In this study, the new fluorescence-based POC system with estimated material cost of 3.000 $: a ten times lower than the cost of the commercial available is suitable for resource-limited setting for rapid, sensitive, and low-cost pathogen diagnostic.

## 5 Conclusion

In conclusion, we developed and successful proved a concept for a new fluorescence-based POC system for rRT-LAMP. The fPOC is optimized by using different excitation LED light sources, filters, and implemented with Op-amp. The performance of the developed fPOC system for rRT-LAMP was tested, evaluated, and compared with another commercial PCR thermocycler Mx3005P, and PATHPOD system, an in-house developed turbidity detection LAMP based POC system. To detect SARS-CoV-2 in clinical samples, the newly developed fPOC system was used, and the results revealed that the performance of the fPOC system is comparable to the commercial PCR thermocycler Mx3005P system. Moreover, the special design of the fPOC with pyramid optical structures to reflect the light into four different reaction wells, as well as the location of all the integrated components on the top of the reaction well, will reduce the number of the light sources needed and enhance the temperature transfer into the bottom of the reaction and open more opportunity for integrated other functions or components (for example sample preparation). To conclude, the use of open-source hardware/software commercially available Arduino Platforms can provide a promising potential for quick and low-cost design/fabrication of a POC device tuning to attain required analytical sensitivity comparable to commercial systems.

## Data Availability

The original contributions presented in the study are included in the article/Supplementary Material, and further inquiries can be directed to the corresponding authors.
