# Supplementary material for: Point-of-care system for rapid real-time detection of SARS-CoV-2 virus based on commercially available Arduino platforms
Source: Front Bioeng Biotechnol. 2022 Aug 4;10:917573. doi: 10.3389/fbioe.2022.917573 (PMC9385952; doi:10.3389/fbioe.2022.917573)
Supplement: Supplementary file 1 [file datasheet1.pdf]

Supplementary Information

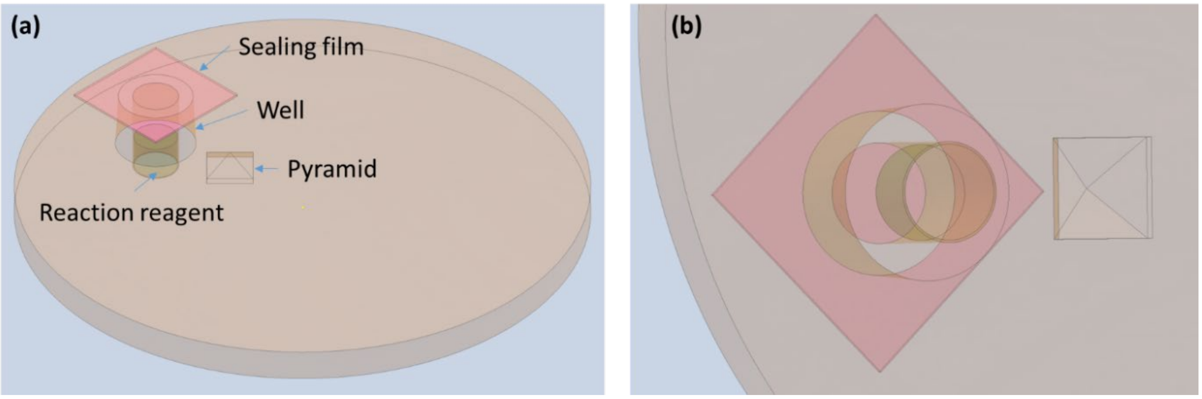

Figure S1. 3D image of the chip with single well (a) op view and (b) bottom view.

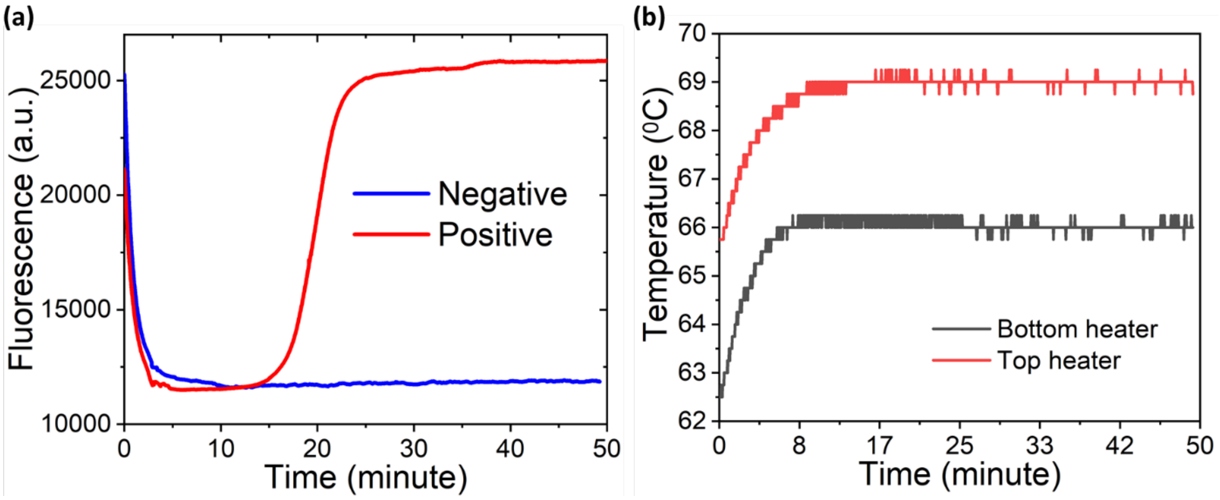

Figure S2. RT-rLAMP experiment on fPOC system: (a) Fluorescence signal intensity with control negative and positive samples, and (b) Controlling temperature of top and bottom heater during reaction.

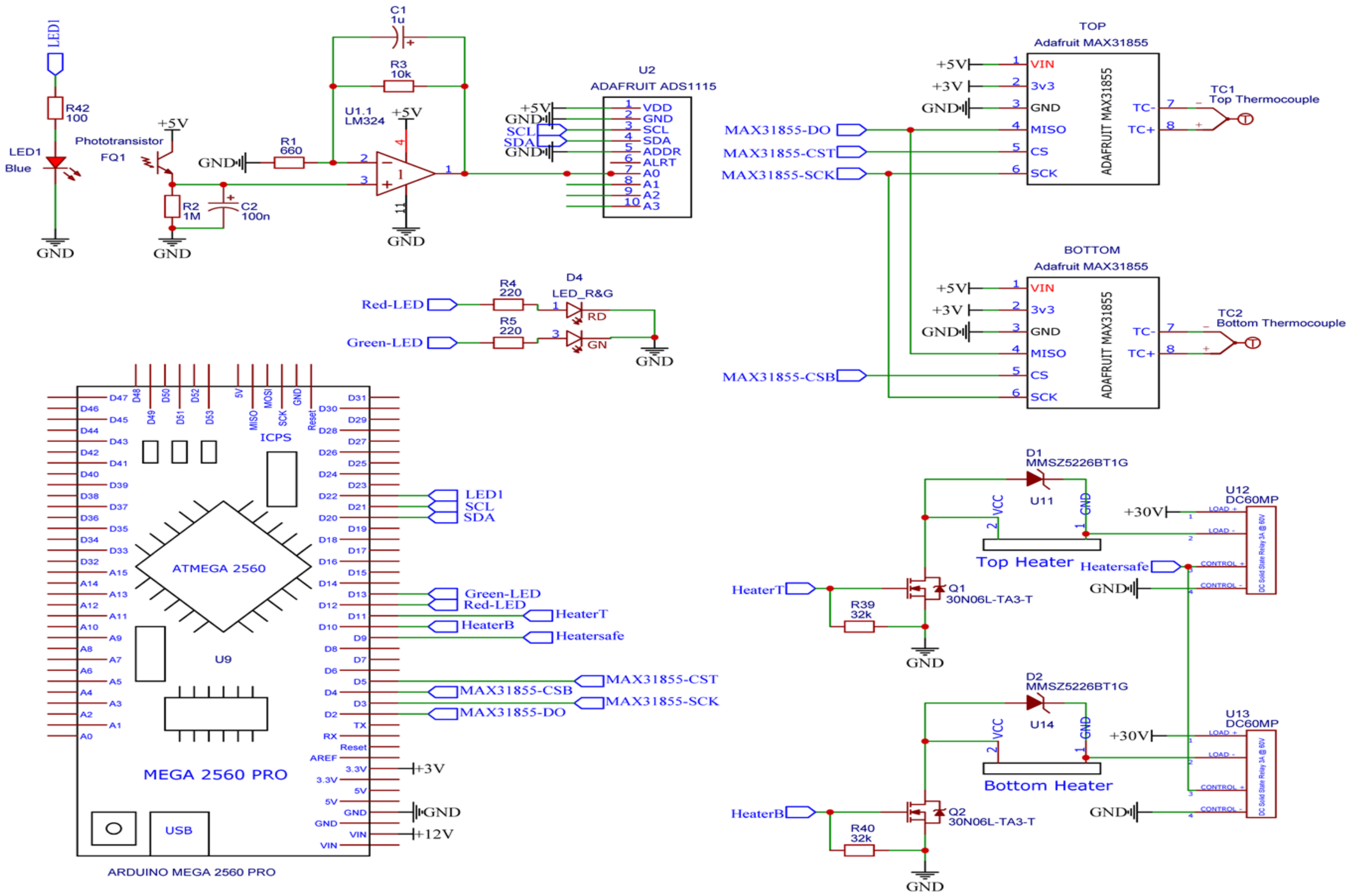

Figure S3. System electronic schematic of the fPOC system with a single sample detection.

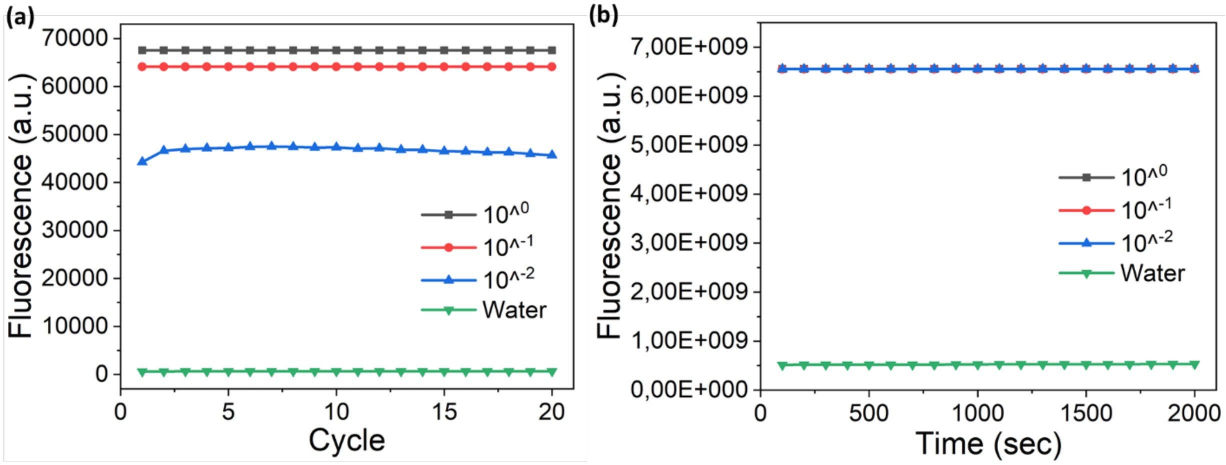

Figure S4. Compare with other qPCR systems: (a) PIKO system-gradient concentration of template at 65 ^{0}C, and (b) gradient with template with qPCR system Mx3005P

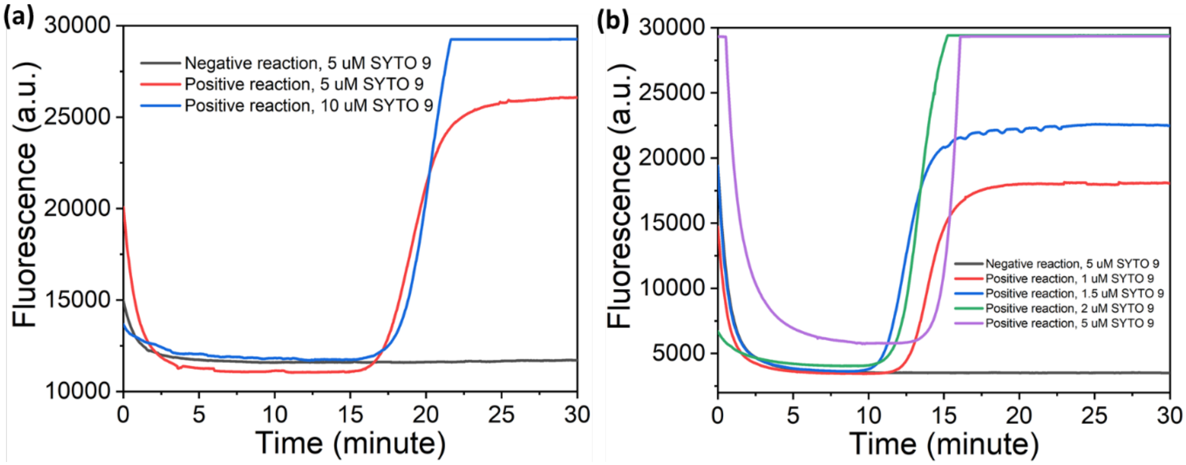

Figure S5. rRT-LAMP reaction on Fluorescence system with different CYTO 9 concentration: (a) Omega filter set, and (b) Optolong Optics filter set.

Table S1. Bill of materials and suppliers

fPOC system component list				
No.	Item name	Supplier	Item number	Price (USD)
1	Controllerboard MEGA 2560	www.mouser.dk	A000067	33.85
2	Adafruit MAX31855	www.mouser.dk	485-269	14.95
3	Adafruit ADS1115 16-Bit ADC	www.mouser.dk	485-1085	14.95
4	Top and Bottom heaters	www.keenovo.com		61.06
5	5TC-TT-KI-30-1M Thermocouples	dk.omega.com	5TC-TT-KI-30-1M	20.31
6	DC60MP - Solid State Relay	dk.farnell.com	7229124	28.63
7	FQP30N06L MOSFET	dk.rs-online.com	807-5863	3.20
8	Vishay BPW77NA, Photo Transistor	dk.rs-online.com	815-1250	5.05
9	Blue LED, C503B-BCN-CV0Z0461	dk.farnell.com	1855508	0.20
10	Blue LED, L-19804VBC/DS-D	dk.farnell.com	2373493	1.07
11	Blue LED, NSPB500AS	dk.rs-online.com	713-3964	1.41
12	Blue LED, HLMP-CB1B-XY0DD	dk.rs-online.com	769-3533	1.40
13	Blue LED, C503B-BAN-CZ0A0451	dk.rs-online.com	123-8663	0.41
14	480 nm filter, CWL:480±5nm, FWHM:30±5nm	www.optolong.com		47.00
15	530 nm filter, CWL:530±5nm, FWHM:30±5nm	www.optolong.com		47.00
16	510 nm filter, CWL:510±2nm, FWHM:10±2nm	www.omegafilters.com	510DF10	60.00
17	465 nm filter, CWL:465±2nm, FWHM:10±2nm	www.omegafilters.com	465DF10	60.00
18	AC / DC Power Supply, 150 W, 30 V, 5 A	dk.farnell.com	2771483	68.41
19	JSM1024S05 DC / DC Converter	dk.farnell.com	2545403	28.35
20	R-78B12-2.0 DC / DC Converter	dk.farnell.com	2747529	13.54
21	Schottky diode rectifier	dk.farnell.com	2675408	0.17
22	Resistance 100 ohm	dk.farnell.com	2614368	0.30
23	Resistance 660 ohm	dk.rs-online.com	683-3941	0.11

24	Resistance 10 kohm	dk.farnell.com	9341110	0.06
25	Resistance 32 kohm	dk.farnell.com	9467297	0.11
26	Resistance 1 Mohm	dk.farnell.com	2330049	0.11
27	Capacitor, 1µF, 63V dc	dk.rs-online.com	181-5470	0.13
28	Ceramic Disc & Plate Capacitor, 0.1 µF, 50 V	dk.farnell.com	1141775	0.18
**Total cost**				**511.96**

Note: Prices were quoted in Denmark on 16th of June 2022

Table S2. Different blue LEDs

LED name	Wavelegth (nm)	Angle (Deg)	Max luminescence (cd)	Current (mA)
C503B-BCN-CV0Z0461	470	30	4.1	20
L-19804VBC/DS-D	470	12	9	20
NSPB500AS	470	15	9.3	20
HLMP-CB1B-XY0DD	470	15	9.6	20
C503B-BAN-CZ0A0451	470	15	23.5	30

Table S3: C_t and T_t values of clinical samples used in this study

No. samples	rRT-PCR (C_t)	fPOC (T_t)	qPCR system (T_t)	PATHPOD (T_t)
1	10.33	14.38	9.93	21.69
2	11.78	14.83	10.21	21.05
3	20.68	15.13	13.22	24.22
4	26.11	16.5	13.21	24.22
5	13.32	14.83	11.55	22.32
6	12.59	14.58	10.99	21.05
7	12.44	13.22	11.41	21.69
8	No C_t	No T_t	No T_t	No T_t
9	No C_t	No T_t	No T_t	No T_t
10	No C_t	No T_t	No T_t	No T_t
11	No C_t	No T_t	No T_t	No T_t
12	No C_t	No T_t	No T_t	No T_t
